# High expression of ABL2 promotes gastric cancer cells migration, invasion and proliferation via the TGF-β and YAP signaling pathways

**DOI:** 10.7150/jca.99307

**Published:** 2024-09-09

**Authors:** Yun Liu, Tao Jin, Ruiyun Chen, Renjie Miao, Yong Zhou, Shihe Shao

**Affiliations:** 1 Department of Gastroenterology, Institute of Digestive Disease, The Affiliated People's Hospital, Jiangsu University, Zhenjiang, Jiangsu, China.; 2 School of Medicine, Jiangsu University, Zhenjiang, Jiangsu, China.; 3 Department of Gastroenterology, Yixing people's hospital, Yixing, Jiangsu, China.; 4 Department of gastrointestinal surgery, Qingdao Central Hospital, University of Health and Rehabilitation Sciences, Qingdao, Shandong, China.; 5 Department of Clinical laboratory, Affiliated Third Hospital of Zhenjiang to Jiangsu University, Zhenjiang, Jiangsu, China.

**Keywords:** gastric cancer, ABL2, epithelial mesenchymal transformation, proliferation, metastasis, invasion.

## Abstract

**Background:** The Abelson-Related Gene (ABL2) is expressed in various malignancies. However, its role in gastric cancer (GC) regarding tumor proliferation, metastasis, and invasion remains unclear.

**Methods:** ABL2 expression in clinical specimens was assessed using quantitative real-time fluorescence PCR (qRT-PCR). Western blotting and immunofluorescence assay determined protein levels. Additionally, Transwell migration and invasion, cell counting kit-8 (CCK-8) and colony-formation assays analyzed the effect of ABL2 on GC cells. Protein levels related to GC cells were assessed through Western blotting. The effects of si-ABL2 combined with GA-017 that activated YAP on cell migration, invasion and proliferation were investigated.

**Results:** ABL2 expression was upregulated in human GC tissues compared to paracancer tissues, and it was positively related to tumor node metastasis classification (TNM) stage. Furthermore, high ABL2 levels promoted the proliferation, metastasis, and invasion capacity in GC cells. Elevated ABL2 expression enhanced the expression of MMP2, MMP9, and PCNA while decreasing TIMP1 and TIMP2 expression. It also increased the p-SMAD2/3 expression and YAP expression, decreased the expression of p-YAP in GC cells. Furthermore, GA-017 increased ABL2 expression in MGC-803 cells and counteracted the effects of si-ABL2 on cell migration, invasion and proliferation.

**Conclusion:** These findings indicated that heightened ABL2 expression could activate TGF-β/SMAD2/3 and YAP signaling pathway, promoting epithelial mesenchymal transformation (EMT), and enhancing multiplication, metastasis, and invasion in GC cells.

## 1. Introduction

Gastric cancer (GC) stands as the third most lethal malignancy globally, while ranking fifth in frequency 1, 2. Its multifactorial and complex nature involves regional environmental and dietary factors. Notably, *Helicobacter pylori* (*H. pylori*) infection, race, ethnicity, age and sex are all risk factors associated with GC risk 3, 4. Therefore, investigating the molecular mechanism driving GC progression offers a new avenue for advancing its diagnosis and treatment.

The ABL family genes, particularly non-receptor tyrosine kinases ABL1 (c-ABL) and ABL2 (Arg), play an essential role in various biological processes, including cell proliferation and division 5-7. Similarly, Zhang Y *et al.* reported that ABL2 was upregulated in cervical cancer (CC) tissues. Additionally, reduced ABL2 expression decreased the migration and invasion capabilities of HepG2 cells, with the mediation of circ_0000263/miR-117 targeting ABL2 implicated in CC progression 6. Qin X *et al.* found that ABL2 downregulation had tumor-suppressive effects on cervical carcinoma proliferation and migration 8. Research indicated an increase in ABL2 levels in renal cell carcinoma (RCC) samples, with higher ABL2 expression correlating with poorer RCC overall survival in the TCGA database for patients with RCC 9. Elevated ABL2 expression has been associated with the inhibition of cell apoptosis in GC 10. These suggested that ABL2 usually acts as a cancer-promoting gene in cancers, including GC, CC and RCC. Nevertheless, there are no relevant reports on whether ABL2 regulates metastasis, invasion and proliferation of GC cells.

Transforming growth factor-β (TGF-β)/SMAD signaling pathway is considered in inducing EMT and metastasis in GC cells 11-13. Also, YAP was expressed in gastric adenocarcinoma tissues, elevating with the ascending order of tumor malignancy, which was associated with the proliferation and metastasis of GC cells 14, 15. The role of TGF-β and YAP signaling pathways in the regulation of metastasis, invasion and proliferation of GC cells by ABL2 has not been studied. Therefore, this study aims to elucidate the function of ABL2 in GC specifically focusing on its impact on cell proliferation, migration, and invasion through the YAP signaling pathway.

## 2. Material and methods

### 2.1 Human tumor tissues

We collected 36 tissue samples from 36 patients with GC from the Affiliated People's Hospital, Jiangsu University. In this study, paracancer tissues refer to the tissue located 2 cm away from the tumor edge. We obtained both GC tissues (T) and corresponding paracancer tissues (P) for analysis. The participants were randomly selected from individuals with confirmed GC diagnoses. The following inclusion criteria were used: (1) patients who did not undergo any form of anti-tumor therapy such as radiotherapy and chemotherapy before surgery; (2) patients with a confirmed GC diagnosed through pathological diagnosis. The exclusion criteria were as follows: (1) presence of a family history of GC; (2) prior radiotherapy and chemotherapy; (3) metastasis from other malignant tumors. All participants provided informed consent, and the study was approved by the Ethics Committee of the Jiangsu University (Zhenjiang, China) in May 2017 (K-20170105-Y). The GC patients included 13 females and 23 males; the median age of patients was 69.31 ± 7.179 years. Detailed clinicopathological data of these GC patients are shown in Table 1.

### 2.2 Cell culture

We obtain human undifferentiated GC cell lines HGC-27 and the normal human gastric epithelial cells GES-1 from the Cell Bank of the Chinese Academy of Sciences (Shanghai, China). Human gastric poorly differentiated mucoid adenocarcinoma derived cells MGC-803 cells were obtained from the American Type Culture Collection (ATCC, Manassas, VA). Cells were cultured in RPMI 1640 medium (Gibco, Thermo Fisher Scientific) supplemented with 10% FBS (Gibco, Thermo Fisher Scientific, Waltham, MA) and maintained at 37°C in a 5% CO_2_ atmosphere-involving.

### 2.3 Overexpression vector, small interfering RNA, and cell transfection

Si-ABL2 (si-ABL2-1: sense: 5'-GCAGUAGUCCAGAAGCUUUTT-3', antisense: 5'-AAAGCUUCUGGACUACUGCTT-3'; si-ABL2-2: 5'-GCUGGAGCCAAAUUUCCUATT-3', antisense: 5'-UAGGAAAUUUGGCUCCAGCTT-3') and negative control (NC) were designed by Gene pharma Biotechnology (Suzhou, China). Cell transfection used with Lipofectamine^TM^ 2000 (Thermo Fisher Scientific lnc., USA) and performed following the manufacturer's instructions. The Arg Lentiviral Activation Particles (sc-417779-LAC) and vector particles were obtained from Santa Cruz (Santa Cruz Biotechnology, Santa Cruz, CA, USA). Then, 1×10^4^ HGC-27 cells were seeded into 24-well cell culture plate; next day, the Arg Lentiviral Activation Particles and vector particles infected HGC-27 cells with multiplicity of infection = 25. Additionally, protamine sulfate salt from salmon (also called salmine) was obtained from Sigma-Aldrich (Hong Kong, China) and used as a substitute for polybrene to facilitate the lentivirus infection process. Furthermore, puromycin (Biosharp, Hefei, China) was used for the selection of HGC-27 cells stably over-expressing ABL2.

### 2.4 RNA extraction and quantitative real-time polymerase chain reaction (qRT-PCR) analysis

Total RNA was extracted from tissue samples using TRIzol Reagent (Invitrogen, Life Technologies Corporation), according to the manufacturer's protocol. RNA was reverse transcribed using HiScript QRT SuperMix from the qPCR Kit (Vazyme, Nanjing, China). qRT-PCR amplification was conducted using TransStart Top Green qPCR Super Mix (TRAN, China) on an ABI Step One Plus Real-Time PCR System (Applied Biosystems, Foster City, CA, USA). Data was analyzed using the 2^-ΔΔCT^ method and normalized using GAPDH expression in each sample.

### 2.5 Western blotting assay

Cellular and tissue protein extraction was performed using 1% phenylmethanesulfonyl fluoride (PMSF)-containing buffer for radioimmunoprecipitation (RIPA) assay (Beyotime Biotechnology, Shanghai, China). Protein concentration was determined using a Nandrop1000 spectrophotometer (Thermo Fisher, USA). Subsequently, 20 μg of the cell proteins were separated on 10-well SDS-PAGE gels, and 120 μg of proteins were separated on 15-well gels, followed by an electrophoretic transfer onto PVDF membranes. Following this, membranes were blocked with dry skimmed milk (5%), and primary antibodies were incubated overnight at 4°C (Table 2). The following day, membranes were incubated for an additional 1 h with HRP-conjugated anti-rabbit IgG (H+L) goat secondary antibodies (Fcmcs, Nanjing, China) at ambient temperature. After three washes, an enhanced chemiluminescence system (Image Quant LAS 4000) was used to visualize the membranes. All assays were performed in triplicates. Image J software was used to quantitatively analyze the protein bands.

### 2.6 Transwell migration and invasion assay

Eight-μm-pore size Costar Transwell chambers (Corning, Costar, NY, USA) were used to analyze the cell migration and invasion ability. For cell migration assay, the upper compartments were filled with serum-free medium (200 μL) containing 2×10^4^-5×10^4^ cells, while the lower compartments were filled with a 10% FBS-containing medium (600 μL). Cells were incubated at 37°C in a 5% CO_2_ atmosphere for 24 hours, fixed with 4% paraformaldehyde, and stained with crystal violet. After imaging, cell counting was performed using an optical microscope (Olympus, Germany) in five random areas of transwell chambers. Before cell invasion assay, Corning BioCoat Matrigel was diluted eightfold with serum-free medium, and 40 μL was added to the upper chamber, which was then solidified at 37°C for 30 min, other experimental procedures are the same as cell migration assay.

### 2.7 The colony-formation and cell proliferation assay

The assays were performed using CCK-8 (Tongren, Shanghai, China) and colony-formation kits to assess the impact of ABL2 on the proliferation capacity of MGC-803 cells and HGC-27 cells. A 1000 transfected cells and NC cells were seeded into 96-well plates. The next day, each well was treated with CCK-8 solution (10 μL) and incubated for 1 h. A microplate reader was used for absorbance (450 nm) measurements at different times (continuous testing for three days). Another 1000 cells were seeded in six-well plates and cultured at 37°C in a 5% CO**_2_** atmosphere. Medium (10% FBS) was replaced every three days until visible cell clones formed in six-well plates after 10-14 days. The cell clones were then fixed with paraformaldehyde (4%) fixation and stained with crystal violet.

### 2.8 Immunofluorescence assay

The cells were seeded on glass coverslips and then cultured at 37°C in a humidified incubator with 5% CO_2_ for 24 h. Subsequently, the cells were fixed with 4% paraformaldehyde for 30 min at room temperature. Cell membranes were permeabilized with 0.5% TritonX-100 (Sigma-Aldrich) for 20 min at room temperature and blocked with 5% bovine serum albumin (BSA) for 30 min. Glass coverslips were incubated with primary antibodies overnight at 4°C. The following day, the slides were washed and incubated with FITC-labeled Goat Anti-Rabbit IgG (H+L) (Beyotime, Guangdong, China) diluted at 1:300 at room temperature for 30 min. Finally, the slides were stained with 1µg/ml Hoechst 33258 nuclear stain (Sigma-Aldrich, Hong Kong, China) for 5 min at room temperature and mounted with Antifade Mounting Medium (Beyotime). Images were acquired using confocal laser scanning microscopy.

### 2.9 Statistical analysis

The GraphPad Prism 8.2 statistical software (La Jolla, USA) was used for statistical analysis. The independent t-test was used for comparisons among groups. CCK-8 results were analyzed using a Two-way ANOVA test. The correlation between the expression level of ABL2 and clinicopathological parameters in GC tissues was analyzed using Pearson's chi-squared test and Fisher's exact test. Each experiment was repeated thrice. A *P*-value < 0.05 was considered statistically significant.

## 3. Results

### 3.1 ABL2 is highly expressed in GC tissues and cells

**Figure 1:**
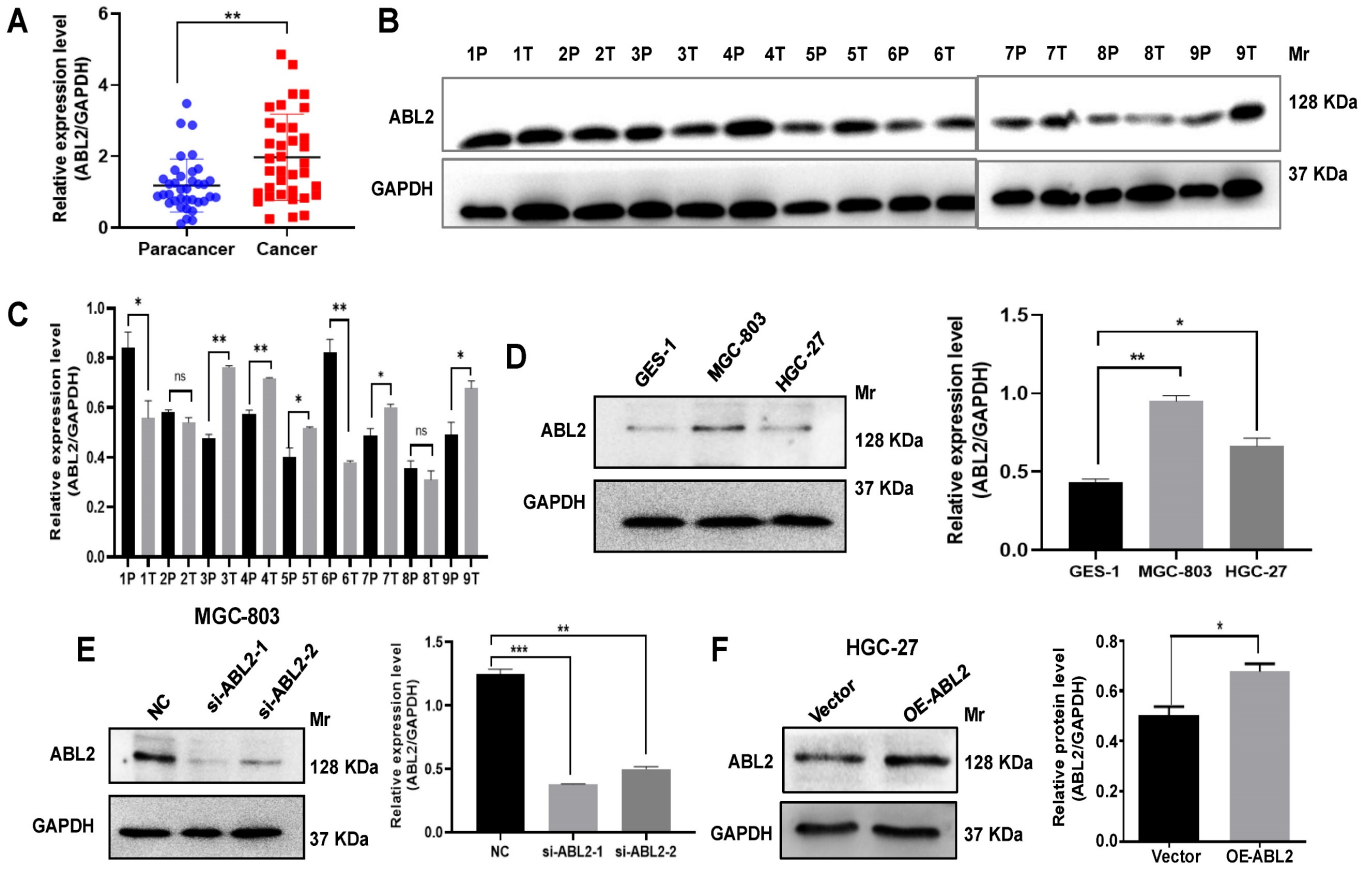
**ABL2 is overexpressed in GC tissues and cell lines. (A)** ABL2 mRNA expression in 36 paired GC tissues and the corresponding paracancer tissues was detected by qRT-PCR. **(B)** Representative image shown detection of ABL2 expression in GC tissues (T) and corresponding paracancer tissues (P) from 9 GC patients by Western blotting assay. **(C)** Quantitative analysis of the relative ABL2 expression level in GC tissues. **(D)** Left, Representative image shown detection of ABL2 expression in GES-1 cells, MGC-803 cells and HGC-27 cells by Western blotting assay. Right, Quantitative analysis of the relative ABL2 expression level. **(E)** Left, Representative image shown detection of ABL2 expression in MGC-803 cells was knocked down by transfecting si-ABL2. Right, Quantitative analysis of the relative ABL2 expression level. **(F)** Left, Representative image shown detection of ABL2 expression by using Arg Lentiviral Activation Particles to overexpress ABL2 in HGC-27 cells. Right, Quantitative analysis of the relative ABL2 expression level. Data are presented as the mean ± SD of 3 independent experiments. *P<0.05, **P<0.01, ***P<0.001. *Mr*: Molecular size.

To evaluate the role of ABL2 in GC processing, we first investigated ABL2 expression level in the GC tissues. Our qRT-PCR analysis revealed high ABL2 expression in GC tissues that compared to paracancer tissues (Fig.1A), and ABL2 expression was closely associated with TNM stage, but no significant difference between sex, age tumor size, differentiation, lymph node metastasis (LNM) and distance metastasis (Table 1). Western blotting assay results shown that ABL2 expression increased in five out of nine GC patient tissues (Fig. 1B and C). In addition, to determine the expression of ABL2 in gastric mucosal cells, we using normal human gastric epithelial cells (GES-1 cells), human undifferentiated GC cell line (HGC-27), and human gastric poorly differentiated mucoid adenocarcinoma derived cells (MGC-803 cells), detected ABL2 expression level in the different cell line or cells. As shown in Fig.1D, ABL2 expression was particularly higher in MGC-803 cells and lower in HGC-27 cells.

Next, we tried to investigate down or up regulation of ABL2 expression, two siRNAs (si-ABL2-1 and si-ABL2-2) were used to knock down ABL2 expression in the MGC-803 cells and the Arg Lentiviral activation Particles was used to manipulate the expression level of ABL2 in HGC-27 cells. The expression of ABL2 was knocked by transfecting both of si-ABL2-1 and si-ABL2-2 in MGC-803 cells (Fig.1E). ABL2 expression level significantly enhanced by using Arg Lentiviral activation Particles to overexpress that compared to vector particles-infected HGC-27 cells (Fig.1F). Taken together, these data indicate that ABL2 is highly expressed in GC tissues and cell lines, the ABL2 expression level was downregulated by si-RNA, or upregulated by Arg Lentiviral activation Particles for overexpression.

### 3.2 ABL2 expression influenced cell proliferation, migration, and invasion *in vitro*

**Figure 2:**
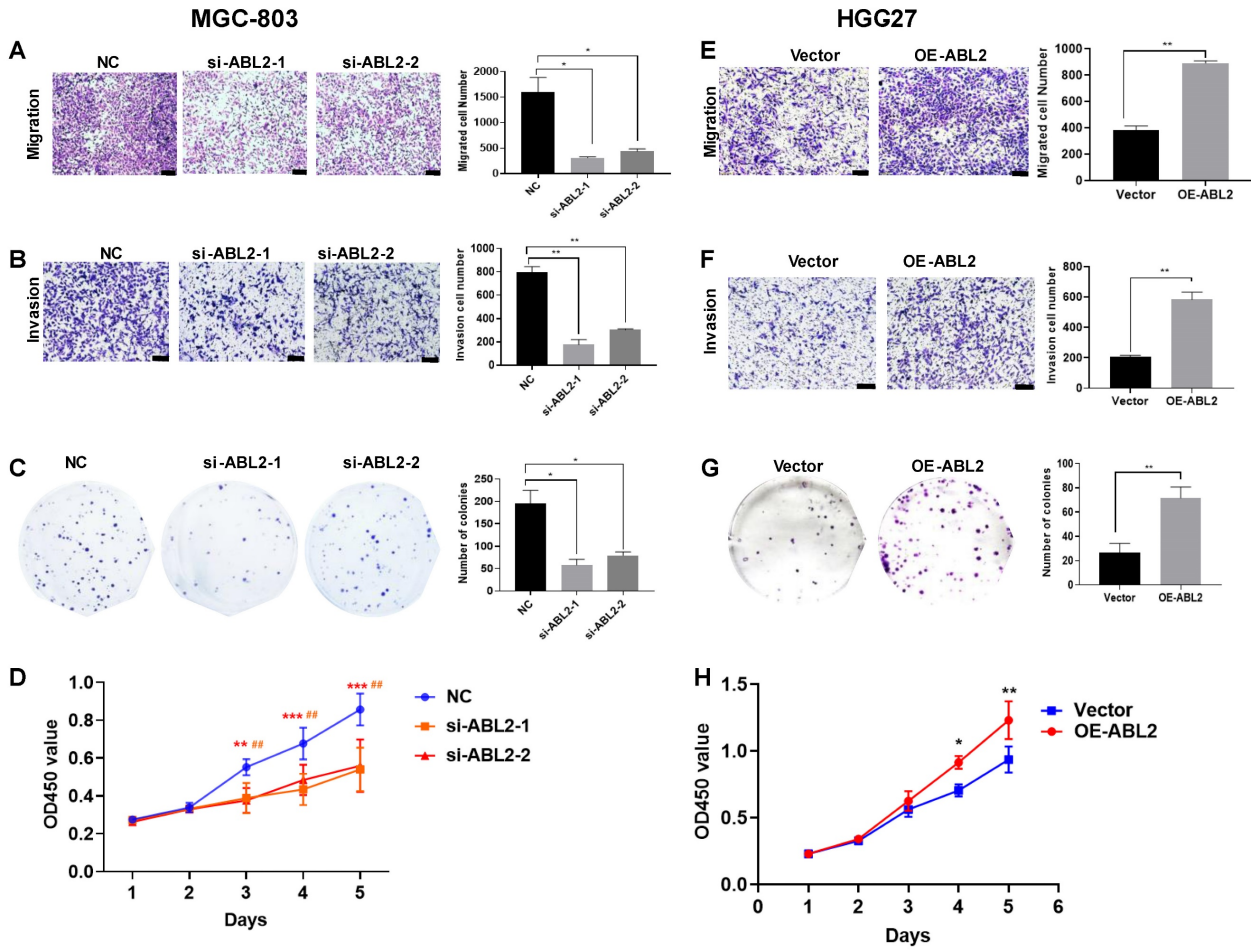
** High expression of ABL2 promoted cell migration, invasion and proliferation in GC cells. (A)** Left, Images of trans-membrane cells in the NC and si-ABL2 group of MGC-803 cells (scale bars = 200 μm). Right, Quantitative analysis of trans-membrane cells; **(B)** Left, Images of invading membrane cells in the NC and si-ABL2 group of MGC-803 cells (scale bars = 200 μm). Right, Quantitative analysis of invading membrane cells; **(C)** Left, Images of cell colonies in the NC and si-ABL2 group of MGC-803 cells. Right, Quantitative analysis of cell colonies. **(D)** CCK-8 assay detected the effects of low expression of ABL2 on cell growth ability of MGC-803 cells. **(E)** Left, Images of transmembrane cells in Vector and OE-ABL2 groups HGC-27 cells (scale bars = 200 μm). Right, Quantitative analysis of transmembrane cells; **(F)** Left, Images of invading membrane cells in Vector and OE-ABL2 groups HGC-27 cells (scale bars = 200 μm). Right, Quantitative analysis of invading membrane cells; **(G)** Left, Images of cell colonies in Vector and OE-ABL2 groups HGC-27 cells. Right, Quantitative analysis of cell colonies. **(H)** CCK-8 assay examined the effect of high ABL2 expression in HGC-27 cells on cell growth ability when compared with the Vector group. Data are presented as the mean ± SD of 3 independent experiments. *P<0.05, **P<0.01, ***P<0.001; ^##^*P*<0.01, in CCK-8 si-ABL2-2 vs NC group.

To determine whether ABL2 expression level contribute to cell proliferation, migration, and invasion *in vitro*, we performed Transwell migration and invasion assays, colony-formation and CCK-8 assays in MGC-803 and HGC-27 cells. The results demonstrated that ABL2 knockdown significantly reduced the number of migrated and invaded MGC-803 cells (Fig. 2A and B). The colony-formation assay revealed that ABL2 knockdown resulted in the formation of smaller and fewer clones (Fig. 2C). The CCK-8 assay found that knockdown of ABL2 decreased the OD450 value of GC cells compared to control cells, with an effect on cell growth starting on the third day for MGC-803 cells (Fig. 2D). Conversely, overexpression of ABL2 in HGC-27 cells promoted cell migration and invasion (Fig. 2E and F), increased cell proliferation (Fig. 2G), with high expression influencing cell growth in days 4 and 5 (Fig. 2H).

### 3.3 ABL2 expression level related to cell proliferation, migration and epithelial mesenchymal transformation (EMT)

**Figure 3:**
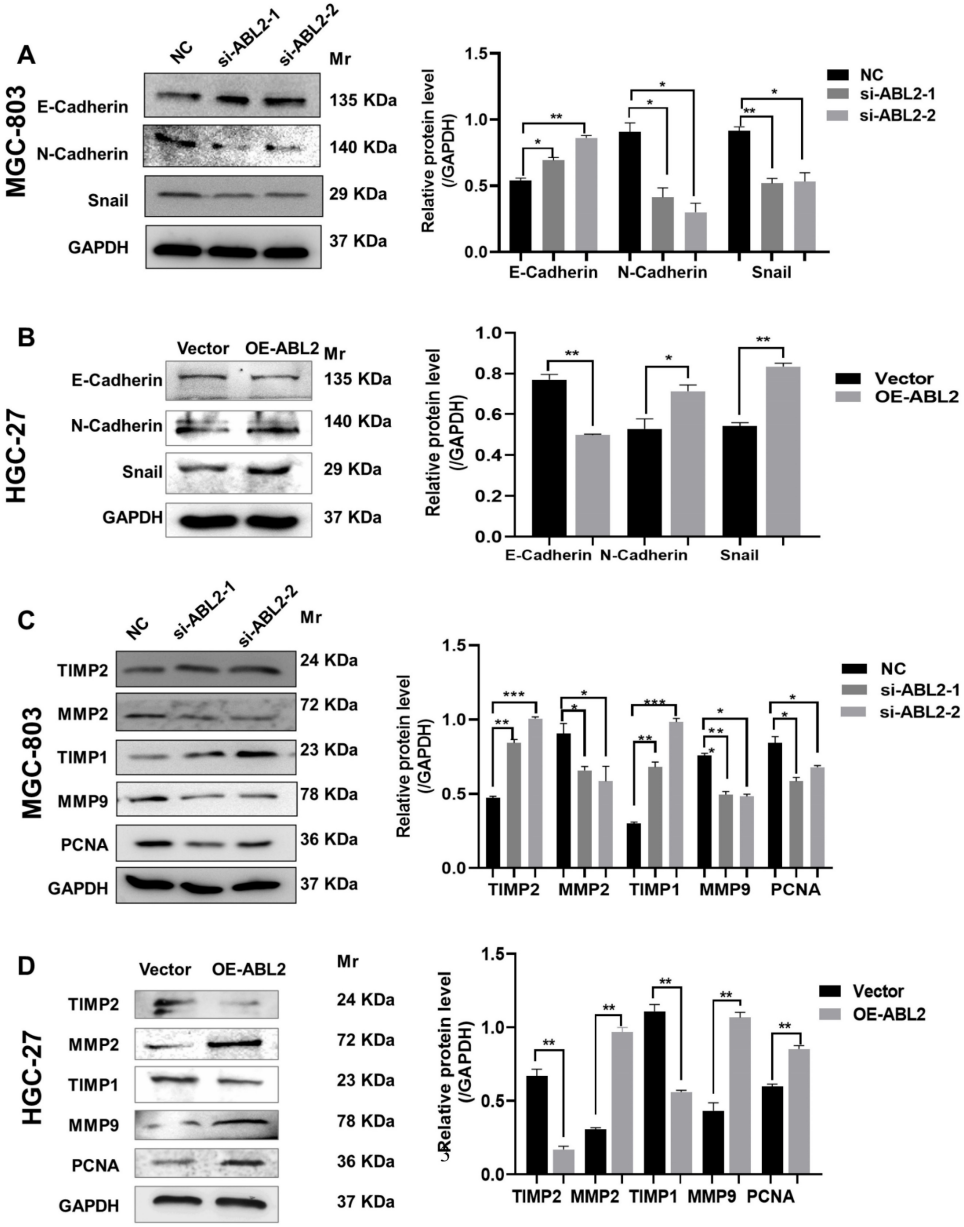
** Knocking down ABL2 expression decreased the protein levels related to migration, proliferation, and EMT in GC cells. (A)** Left, Representative image shown the expression of E-Cadherin, N-Cadherin and Snail in MGC-803 cells transfected with NC and si-ABL2. Right, Quantitative analysis of EMT-related proteins' relative expression levels. **(B)** Left, Representative image shown the expression of E-Cadherin, N-Cadherin and Snail in HGC-27 cells transfected with the Arg Lentiviral Activation Particles and Vector. Right, Quantitative analysis of EMT-related proteins' relative expression levels. **(C and D)** Left, Representative image shown the expression of MMP2, MMP9, PCNA, TIMP1 and TIMP2 in MGC-803 cells **(C)** or HGC-27 cells **(D)** with knocked down or overexpressed ABL2 expression. Right, Quantitative analysis of migration- or proliferation- proteins' relative expression levels. Data are presented as the mean ± SD of 3 independent experiments. *P<0.05, **P<0.01, ***P<0.001.

Cadherins are membrane-spanning glycoproteins that couple cell-cell adhesion to the cytoskeleton and play an important role in tissue homeostasis. Different expressions of E-cadherin or N-cadherin related to human cancers as well as in animal models. Loss of E-cadherin expression during EMT is associated with tumor development 16, 17. Snail plays an important role in embryonic development and cancer progression as a key transcriptional repressor of E-cadherin expression in EMT 18. Therefore, we asked whether ABL2 knockdown might alter E-cadherins, N-cadherins, or Snail expressions that are closely connected with tumorigenesis during EMT. We found that in MGC-803 cells, ABL2 knockdown increased E-cadherin expression and reduced N-cadherin and Snail expressions (Fig. 3A). Conversely, the mesenchymal markers, N-cadherin and Snail were increased in the OE-ABL2 group, whereas the epithelial marker E-cadherin was significantly decreased (Fig. 3B). Thus, these findings suggest that ABL2 overexpression promotes EMT in GC cells *in vitro*.

In addition, matrix metalloproteinases (MMPs) are part of the endopeptidase family and can cleave the majority of components of the extracellular matrix, playing significant roles in the metastasis of malignant tumors 19, 20. Upregulation of MMPs and downregulation of their tissue inhibitors, also named Tissue inhibitor of metalloproteinases (TIMPs), have a close relationship with the invasiveness and metastasis of cancer cells 21. We further explored whether ABL2 influenced the expression of MMPs and TIMPs to regulate cell metastasis and invasion ability. As shown in Fig. 3C, MMP9, MMP2, and PCNA expression were significantly reduced in MGC-803 cells transfected with si-ABL2 to the control cells. TIMP-1 and TIMP-2 expressions were increased. Furthermore, compared to the control cells, MMP-9, MMP-2, and PCNA expressions increased in the OE-ABL2 group of HGC-27 cells, while TIMP-1 and TIMP-2 expressions significantly decreased (Fig. 3D). This suggests that ABL2 expression influences GC cell proliferation and migration-related protein expression *in vitro*.

### 3.4 ABL2 induced GC cell migration, invasion, and proliferation through the TGF-β/SMAD2/3 and YAP signaling pathway

**Figure 4:**
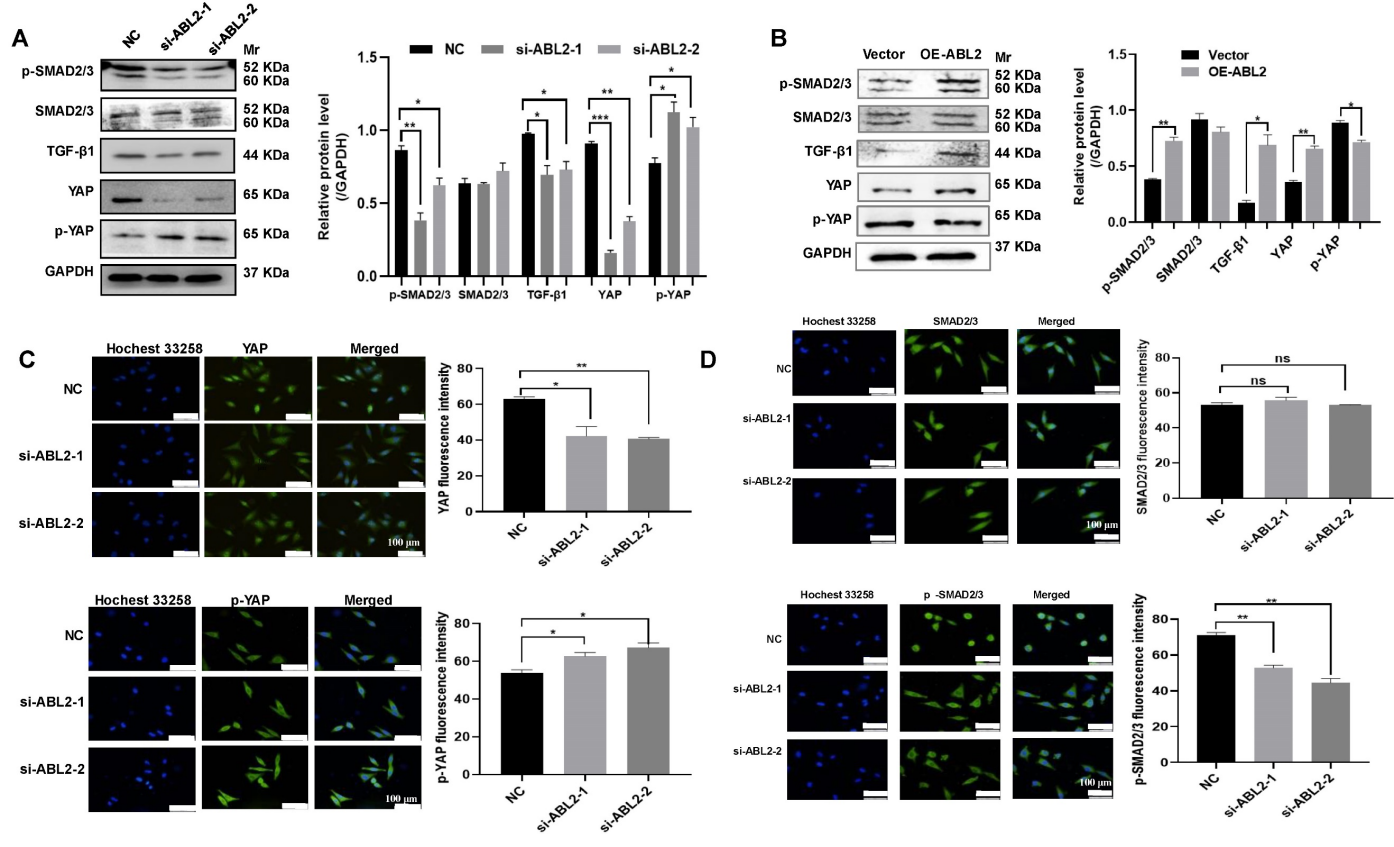
** ABL2 expression affected TGF-β/SMAD2/3 and YAP signaling pathway related proteins. (A and B)** Left, Representative image shown the expression of TGF-β1 and YAP signaling pathway related proteins in MGC-803cells **(A)** and HGC-27 cells **(B)** with knocked down or overexpressed ABL2 expression. Right, Quantitative analysis of the relative protein expression levels. **(C)** Left, Representative image of cell fluorescence showing that knockdown of ABL2 in MGC-803 cells decreases YAP expression and increases p-YAP expression (scale bars = 100 μm). Right, Quantitative analysis of the mean fluorescent intensity levels. **(D)** Left, Representative image of cell fluorescence indicating that knockdown of ABL2 in MGC-803 cells decreases p-SMAD2/3 expression (scale bars = 100 μm). Right, Quantitative analysis of the mean fluorescent intensity levels. Data are presented as the mean ± SD of 3 independent experiments. *P<0.05, **P<0.01, ***P<0.001.

The TGF-β/ SMAD2/3 signaling pathway can affect the metastasis of GC cells 12, 13, and the YAP signaling pathway can affect the ability of metastasis and proliferation of GC cells 15. Thus, we examined TGF-β1, SMAD2/3 and its phosphorylated protein (p-SMAD2/3), as well as YAP and its phosphorylated protein (p-YAP) in GC cells to explore their potential involvement in ABL2-mediated EMT, cell migration and invasion, and proliferation. As shown in Fig. 4B, HGC-27 cells displayed no significant change in SMAD2/3, whereas p-SMAD2/3 increased in the OE-ABL2 group HGC-27 cells compared to the control cells. Additionally, the expression of TGF-β1 increased significantly. In addition, it is speculated that the Hippo signaling pathway plays a significant role in these processes. The level of YAP increased while that of p-YAP decreased in HGC-27 cells overexpressing ABL2 (Fig. 4B). Moreover, knockdown of ABL2 in MGC-803 cells resulted in reduced expressions of TGF-β1, p-SMAD2/3, and YAP, while increasing p-YAP expression (Fig. 4A). Immunofluorescence analysis also revealed that knockdown of ABL2 in MGC-803 cells decreased YAP and p-SMAD2/3 expression but increased p-YAP expression (Fig. 4C and D).

**Figure 5:**
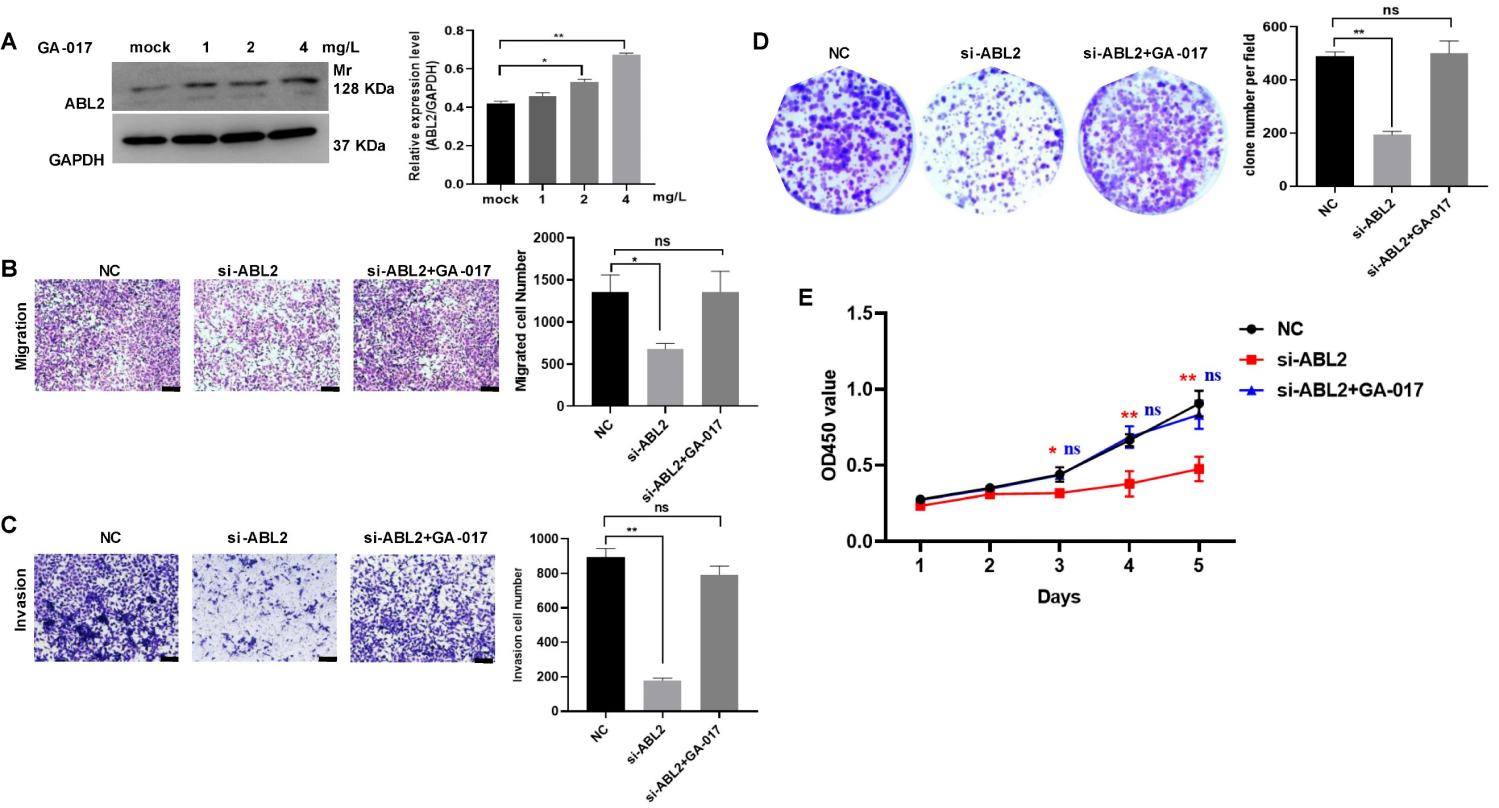
**The effect of YAP/TAZ activation GA-017 in GC cells. (A)** Left, Representative image shown the expression of ABL2 in MGC-803cells treated with GA-017 was detected using Western blotting. Right, Quantitative analysis of the relative protein expression levels. **(B)** Left, Representative image of trans-membrane cells in NC, si-ABL2 and si-ABL2 treated with GA-017 group MGC-803 cells.

Furthermore, GA-017, which promotes YAP/TAZ activation and nuclear translocation, was observed to increase ABL2 expression in MGC-803 cells (Fig.5A). Other assays also revealed that GA-017 can reverse the effects of si-ABL2 on the biological behaviors of MGC-803 cells, such as proliferation, metastasis, and invasion (Fig. 5B-D). It was found that knock down ABL2 has a significant effect on cell grown ability on day 3, 4 and 5, however, GA-017 reversed this effect on day 3, 4 and 5 (Fig. 5E). Collectively, these findings suggest that high ABL2 levels may enhance GC cell migration, invasion, and proliferation by mediating the YAP signaling pathway.

## 4. Discussion

The role and specific implications of ABL2 in GC remain ambiguous despite its investigation in other tumors. Previous studies have delved into ABL2's involvement in GC cell apoptosis, and regulatory mechanisms such as circPGD/miR-16-5p mediation of ABL2 expression 10, 22, its effect on migration, invasion, and proliferation in GC cells has not been thoroughly investigated. In this study, the expression of ABL2 increased in GC tissues and cell lines, and positively correlated TNM stage of GC patients. We observed significant differences in the expression of ABL2 in different patients with GC, which may be attributed to cancer types, *H.pylori* infection, and lymph node metastasis. Therefore, it is crucial to continue collecting and analyzing samples from patients with GC to comprehensively understand ABL2's relation to cancer types or other parameters. High ABL2 expression in HGC-27 cells significantly promoted cell migration, invasion, and proliferation; meanwhile, ABL2 knockdown in MGC-803 cells inhibited these processes.

Moreover, abnormally expressed ABL2 regulates MMP9, MMP2, TIMP1, TIMP2 and PCNA expressions, consequently modulating the migration, invasion, and proliferation of the GC cell lines. EMT plays an essential role in the metastasis and progression of cancer cells; it is a complex biological process wherein cells lose their differentiated epithelial-like state and gain a more mesenchymal-like phenotype. Increased mesenchymal-like phenotype is the early-stage change of cancer 23, 24. E-Cadherin is a predominant epithelial cell marker. Notably, N-Cadherin, Snail, and Vimentin are increased in cancer cells and act as tumor markers 25-27. In the present study, the overexpression of ABL2 upregulated N-Cadherin and Snail but downregulated E-Cadherin in HGC-27 cells. However, further analysis is required to understand the correlation between mesenchymal-epithelial transition and ABL2 in GC cells and to comprehensively explore the role of ABL2 in the metastasis of GC cells.

The TGF-β signaling pathway plays a pivotal role in regulating cell growth, differentiation, motility, invasion, and development in various biological systems 28, 29. Previous studies have demonstrated that the YAP signaling pathway is a vital growth regulator of cell proliferation and apoptosis, further evidenced by the mosaic screens in *Drosophila melanogaster* 30-32. In this study, our observation led us to speculate that ABL2 could regulate GC cell biology through the TGF-β1 and YAP signaling pathways. Herein, the knocking down of ABL2 distinctly inhibited GC cell migration, proliferation, and EMT; conversely, high ABL2 led to the opposite effects. Similarly, the levels of TGF-β1, p-SMAD2/3, and YAP were enhanced in ABL2 overexpressed HGC-27 cells but decreased in MGC-803 cells with knocked-down ABL2 expression.

The main limitation of this study was the inclusion of a small number of patients. Although the preferred treatment of GC in the referral tertiary center is surgery, numerous patients were initially treated with radiation therapy or chemotherapy. Thus, larger cohorts of patients including randomized clinical trials would be essential in understanding the true relevance of these biomarkers in the diagnosis, treatment, and prognosis of GC. Furthermore, analysis using the TGF-β1 signaling pathway-related inhibitor or inducer could provide deeper insights into ABL2's role in promoting GC progression.

The strong expression of ABL2 has been shown to promote GC cell proliferation, migration, and invasion through mediation by the YAP signaling pathway. This suggests its potential as a diagnostic and therapeutic indicator for patients with GC.

## Figures and Tables

**Table 1:** The correlation of ABL2 expression with the clinical features of GC patients.

	Case	High (n=18)	Low (n=18)	*P* value
Gender				0.2979
Male	23	13	10	
Female	13	5	8	
Age				0.6026
≤60	4	3	1	
>60	32	15	17	
Tumor size				0.3166
≤5	19	8	11	
>5	17	10	7	
Differentiation				0.2979
Poor	13	8	5	
Moderate+High	23	10	13	
LNM				
Yes	21	12	9	0.3105
No	15	6	9	
TNM				
Ⅰ+Ⅱ	17	5	12	0.0194*
Ⅲ+Ⅳ	19	13	6	
Distance metastasis				
Yes	17	11	6	0.0951
No	19	7	12	
*P<0.05				

**Table 2:** The origins of antibodies.

Antibody	company	catalogue	dilution ratio
ABL2	Abcam Co., LTD, Cambridge, UK	ab134134	1:1000
GAPDH	Cell Signaling Technology, lnc, Boston, USA	5174S	1:1000
N-Cadherin	Shenyang Wanlei Biotechnology Co., LTD, China	WL01047	1:500
E-Cadherin	Shenyang Wanlei Biotechnology Co., LTD, China	WL01482	1:500
Snail	Shenyang Wanlei Biotechnology Co., LTD, China	WL01863	1:750
TIMP2	Shenyang Wanlei Biotechnology Co., LTD, China	WL01209	1:750
TIMP1	Shenyang Wanlei Biotechnology Co., LTD, China	WL02342	1:750
MMP2	Shenyang Wanlei Biotechnology Co., LTD, China	WL03224	1:300
MMP9	Shenyang Wanlei Biotechnology Co., LTD, China	WL03096	1:750
PCNA	Shenyang Wanlei Biotechnology Co., LTD, China	WL03213	1:400
p-YAP	Cell Signaling Technology, lnc, Boston, USA	4911s	1:800
YAP	Cell Signaling Technology, lnc, Boston, USA	4912s	1:800
P-SMAD2/3	Shenyang Wanlei Biotechnology Co., LTD, China	WL02305	1:500
SMAD2/3	Shenyang Wanlei Biotechnology Co., LTD, China	WL01520	1:750
TGF-β1	Shenyang Wanlei Biotechnology Co., LTD, China	WL02998	1:750

## Abbreviations

GC: gastric cancer
ABL2: abelson-related gene
qRT-PCR: quantitative real-time fluorescence PCR
CCK-8: cell counting kit-8
TNM: tumor node metastasis classification
TGF-β: transforming growth factor-β
EMT: epithelial mesenchymal transformation
*H.pylori*: *Helicobacter pylori*
CC: cervical cancer
RCC: renal cell carcinoma
PMSF: phenylmethanesulfonyl fluoride
RIPA: radio immunoprecipitation
LNM: lymph node metastasis
